# Identification of a novel *PRMD16*::*SKI* fusion gene in T-prolymphocytic leukemia

**DOI:** 10.3389/fonc.2025.1658257

**Published:** 2025-08-26

**Authors:** Marta Brunetti, Hilde Kollsete Gjelberg, Håkon Reikvam, Francesca Micci

**Affiliations:** ^1^ Section for Cancer Cytogenetics, Institute for Cancer Genetics and Informatics, The Norwegian Radium Hospital, Oslo University Hospital, Oslo, Norway; ^2^ Department of Pathology, Haukeland University Hospital, Bergen, Norway; ^3^ K.G. Jebsen Centre of Myeloid Malignancies, Institute of Clinical Science, Faculty of Medicine, University of Bergen, Bergen, Norway; ^4^ Department of Medicine, Haukeland University Hospital, Bergen, Norway

**Keywords:** T-PLL, PRDM16, *SKI*, *PRMD16*::*SKI*, fusion gene

## Abstract

The presence of the *PRDM16::SKI* fusion gene was described, for the first time, in a T prolymphocytic leukemia (T-PLL) patient with a long indolent period and a late development treatment requiring disease. The fusion transcript was detected by RNA sequencing and validated by reverse transcriptase polymerase chain reaction and Sanger/Cycle sequencing. The chimera occurs between exon 1 of the PR/SET Domain 16 (*PRDM16*) gene and exon 2 of the oncogene V-Ski Avian Sarcoma Viral Oncogene Homolog (*SKI*) gene. The finding provides insight into the role of genetic alterations, including fusion genes, in development and progression of T-PLL and may possibly lead to the development of effective and precise targeted therapy for this disease.

## Introduction

T prolymphocytic leukemia (T-PLL) is a rare hematological malignancy characterized by the proliferation of mature lymphoid T-cells, accounting for around 2% of mature lymphocytic leukemia cases (1). T-PLL affects older adults with a median age at diagnosis > 60 years and it is more common in men than women (2, 3). Due to its rarity, the disease incidence and outcome data are limited. Aberrations of chromosome 8, genetic disorders mainly involving T-cell leukemia/lymphoma 1 (*TCL1*) gene family and inactivation of ataxia-telangiectasia mutated (*ATM*) gene are reported to play a role in the pathogenesis of T-PLL (4). Classically, T-PLL patients at diagnosis present an asymptomatic or “inactive” phase, often within 1–2 years until the progression to the symptomatic or “active” phase (5, 6).

A case of T-PLL that progressed to a highly invasive, organ-infiltrating disease was recently reported by Gjelberg and colleagues (7). The patient’s disease followed an unusual course with a 7-year inactive T-cell lymphocytosis phase before progressing to a more aggressive clinical course (7).

To shed light on the pathogenetic mechanisms of this unusual T-PLL, we screened the transcriptome of the cancer cells in search of fusion genes and found a *PRMD16::SKI* chimeric transcript.

## Methods

RNA was extracted from bone marrow aspirate formalin-fixed and embedded in paraffin (sample 1; **Table 1**), from fresh frozen bone marrow (sample 3) and blood cells (sample 4). The extraction was performed as previously reported (8). Two hundred ng (total RNA from sample 3) were sent for high-throughput pair-end RNA-sequencing to the Genomics Core Facility, Norwegian Radium Hospital, Oslo University Hospital (http://genomics.no/oslo/). The software FusionCatcher (9) was used to find fusion transcripts. To validate the presence of the chimeric transcript, a polymerase chain reaction (PCR) amplification followed by cycle (Sanger) sequencing (ThermoFisher Scientific, Waltham, MA, USA) was performed using the primers combination M13-PRDM16-45FW (5’-TCAAGGAGGAGGAGAGAGATTCCG-3’) and M13-SKI-1627-REV (5’- GAGCTCTTTCTCACTCGCTGACA-3’). Sequence analyses were performed on an Applied Biosystems SeqStudio Genetic Analyzer system (ThermoFisher Scientific). The basic local alignment search tool (BLAST) software (https://blast.ncbi.nlm.nih.gov/Blast.cgi) was used for computer analysis of sequence data (10).

**Table 1:** Diagnosis, karyotypic description, immunophenotypes, and fusion transcript identified in four samples from the patient with T-PLL.

Patient	Sample	Diagnosis	Karyotype	Immunophenotype	Fusion gene
1	Sample 1 – 2014, December	clonal T-cell lymphocytosis	NA	CD3+ , CD19/20+, CD4+, CD23+, CD23+	No fusion gene
	Sample 2 – 2022, May	T-PLL	47,XY,del(5)(q11q13),del(11)(q23),der(14)(?),+mar[cp10]/46,XY[1]	CD2+, CD3+, CD4+, CD5+, CD7+, CD26+, CD28+, CD45+, CD45+, CD52+, , CD30-, CD56-, CD57-, TCL1-, TdT-, EBV-CD10-, CD56-, CD99-, and cyTCL1-	NA
	Sample 3 – 2022, June	T-PLL	47,XY,del(5)(q11q13),del(11)(q23),inv(14)(q11q32),+mar[cp4]/47,idem,del(2)(p21),+8,-mar[4]/46XY[2]	NA	*PRDM16::SKI*
	Sample 4 – 2022, October	T-PLL	NA	NA	*PRDM16::SKI*

NA, no available material for test.

## Results

**Figure 1:**
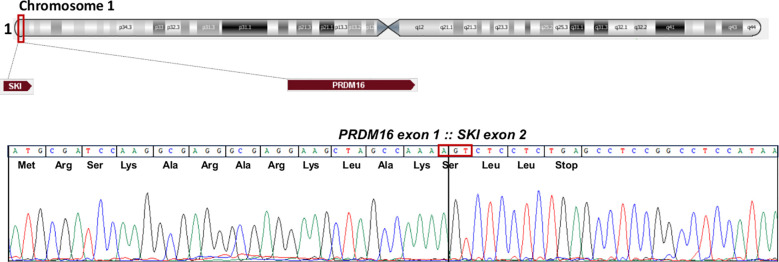
The SKI: PRDM16 fusion transcript. Schematic representation of the *SKI* and *PRDM16* position on chromosome 1. Partial chromatogram showing the junction of the two genes at exons and base pair level, representation of split read mapping *PRDM16::SKI* fusion and predicted sequence of the chimeric protein.

A list of over 400 transcripts was obtained from raw data of sample 3 (data not shown). A specific fusion involving the PR/SET Domain 16 (*PRDM16*) gene and the oncogene V-Ski Avian Sarcoma Viral Oncogene Homolog (*SKI*) was identified as number 27 in the list (spanning unique reads 11). The specific fusion occurs between exon 1 of the *PRDM16* gene (accession number NM_022114.4) and exon 2 of the *SKI* gene (accession number NM_003036.4) (**Figure 1**).

Presence of the *PRDM16::SKI* transcript was then tested in samples 1 and 4. The same fusion was identified in sample 4, but not in sample 1 (**Table 1**). No material to investigate sample 2 was available for the analysis.

## Discussion

We describe, for the first time, the presence of the *PRDM16::SKI* fusion gene in a T-PLL patient. The patient was followed at our institution in the period 2014-2022. Diagnostic analyses were performed on four samples and the cytogenetic findings as well as the immunophenotype are reported in **Table 1**. Patients’ history has been included in a previous publication by Gjelberg et al. (7).

The *PRDM16* gene codes for a zing-finger protein containing a DNA-binding PRDI-BF1/RIZ homologous (PR) domain, and it is commonly rearranged in hematologic malignancies of myeloid lineage, mainly myelodysplastic neoplasms (MDS) and/or acute myeloid leukemia (AML). However, two leukemias of lymphoid lineage have previously been reported, raising the possibility that it could also play a role in lymphomagenesis (11). Survival data suggested a poor prognosis for the patients with AML/MDS and *PRDM16* rearrangements (11).

The *SKI* gene was initially discovered as a viral oncogene, and its over-expression was reported as sufficient for acquiring transforming activity (12). The oncogene *SKI* is a transcriptional co-regulator and seems to contribute to the origin and maintenance of the leukemic phenotype (13). Little is known about its transcriptional regulation during leukemogenesis.

The *PRDM16* and *SKI* gene map both on chromosomal band 1p36 with a distance of 762,438 bp. Such distance is below the resolution level of G-banding and, therefore, could not be seen by this analysis.

The fusion is characterized by an out-of-frame juxtaposition of the genes, with a stop codon coming after 48-base pair. The putative protein is characterized by 12 amino acids from *PRDM16* and three from *SKI* before a stop codon is created. The same fusion was described for the first time by Masetti et al. in a patient with AML harbouring a del(5q), and analysis of *PRDM16* revealed its over-expression (14, 15); additionally it was reported in one AML with a *FLT3-ITD* genetic variant (16). This is the first report in which the *PRDM16::SKI* has been detected in a T-PLL case.

The fact that the fusion was not detected in the initial sample suggested that it may have been acquired in a more advanced phase of the disease. However, genetic investigations of additional samples with an initial indolent clinical course are needed for further conclusions. The occurrence of the fusion gene in both myeloid and lymphoid malignancies is probably more frequent than previously assumed.

The exact mechanism by which *PRDM16::SKI* promotes leukemogenesis is still unknown. However, it has been demonstrated that the short form of PRDM16 (sPRDM16-exon1) promote leukemia development and progression by stimulating cell growth and inhibiting differentiation of AML cells both *in vitro* and *in vivo* (17). It is therefore possible that the leukemogenesis may be may be related to the truncated form of PRDM16 as the breakpoint is between exons 1 and 2. Our patient had three-line treatments, starting with alemtuzumab, followed by venetoclax, and a third-line with combined alemtuzumab and pentostatin (7), and response to therapy was not achieved until the latest line of treatment. However, he developed a quite rapid increase in white blood cell count (WBC) after approximately 15 months. Alemtuzumab and pentostatin were initiated again, inducing a partial response, although due to treatment toxicity and declining general conditions, no further treatment was provided, and the patient died six months later.

In summary, we describe, for the first time, the presence of the *PRDM16::SKI* fusion gene in a T-PLL patient with a long indolent period and a late development treatment requiring disease. It provides further insight into the role of genetic alterations, including fusion genes, in development and progression of T-PLL, and may possibly lead to the development of effective and precise targeted therapy for this disease.

## Data Availability

The original contributions presented in the study are included in the article/supplementary material. Further inquiries can be directed to the corresponding author.

## References

[1] Alaggio R Amador C Anagnostopoulos I Attygalle AD de Oliveira Araujo IB Berti E. Correction: "The 5th edition of the world health organization classification of haematolymphoid tumours: lymphoid neoplasms. Leukemia. (2023) 37:1944–51. doi: 10.1038/s41375-023-01962-5, PMID: 37468552 PMC10457187

[2] Matutes E Brito-Babapulle V Swansbury J Ellis J Morilla R Dearden C. Clinical and laboratory features of 78 cases of T-prolymphocytic leukemia. Blood. (1991) 78:3269–74. doi: 10.1182/blood.V78.12.3269.3269, PMID: 1742486

[3] Vardell VA Ermann DA Fitzgerald L Shah H Hu B Stephens DM. T-cell prolymphocytic leukemia: Epidemiology and survival trends in the era of novel treatments. Am J Hematol. (2024) 99:494–6. doi: 10.1002/ajh.27205, PMID: 38240336

[4] Sun S Fang W. Current understandings on T-cell prolymphocytic leukemia and its association with TCL1 proto-oncogene. Biomedicine Pharmacotherapy. (2020) 126:110107. doi: 10.1016/j.biopha.2020.110107, PMID: 32247279

[5] Braun T von Jan J Wahnschaffe L Herling M. Advances and perspectives in the treatment of T-PLL. Curr Hematol Malig Rep. (2020) 15:113–24. doi: 10.1007/s11899-020-00566-5, PMID: 32034661 PMC7230055

[6] Staber PB Herling M Bellido M Jacobsen ED Davids MS Kadia TM. Consensus criteria for diagnosis, staging, and treatment response assessment of T-cell prolymphocytic leukemia. Blood. (2019) 134:1132–43. doi: 10.1182/blood.2019000402, PMID: 31292114 PMC7042666

[7] Gjelberg HK Helgeland L Liseth K Micci F Sandnes M Russnes HG. Long-smoldering T-prolymphocytic leukemia: A case report and a review of the literature. Curr Oncol. (2023) 30:10007–18. doi: 10.3390/curroncol30110727, PMID: 37999147 PMC10669936

[8] Brunetti M Andersen K Spetalen S Lenartova A Osnes LTN Vålerhaugen H. NUP214 fusion genes in acute leukemias: genetic characterization of rare cases. Front Oncol. (2024) 14:1371980. doi: 10.3389/fonc.2024.1371980, PMID: 38571499 PMC10987735

[9] Nicorici D Şatalan M Edgren H Kangaspeska S Murumägi A Kallioniemi O. FusionCatcher – a tool for finding somatic fusion genes in paired-end RNA-sequencing data. bioRxiv. (2014), 011650. doi: 10.1101/011659

[10] Kent WJ. BLAT–the BLAST-like alignment tool. Genome Res. (2002) 12:656–64. doi: 10.1101/gr.229202, PMID: 11932250 PMC187518

[11] Duhoux FP Ameye G Montano-Almendras CP Bahloula K Mozziconacci MJ Laibe S. PRDM16 (1p36) translocations define a distinct entity of myeloid Malignancies with poor prognosis but may also occur in lymphoid Malignancies. Br J Haematol. (2012) 156:76–88. doi: 10.1111/j.1365-2141.2011.08918.x, PMID: 22050763

[12] Li Y Turck CM Teumer JK Stavnezer E. Unique sequence, ski, in Sloan-Kettering avian retroviruses with properties of a new cell-derived oncogene. J Virol. (1986) 57:1065–72. doi: 10.1128/jvi.57.3.1065-1072.1986, PMID: 3754014 PMC252840

[13] Feld C Sahu P Frech M Finkernagel F Nist A Stiewe T. Combined cistrome and transcriptome analysis of SKI in AML cells identifies SKI as a co-repressor for RUNX1. Nucleic Acids Res. (2018) 46:3412–28. doi: 10.1093/nar/gky119, PMID: 29471413 PMC5909421

[14] Masetti R Togni M Astolfi A Pigazzi M Indio V Rivalta B. Whole transcriptome sequencing of a paediatric case of *de novo* acute myeloid leukaemia with del(5q) reveals RUNX1-USP42 and PRDM16-SKI fusion transcripts. Br J Haematol. (2014) 166:449–52. doi: 10.1111/bjh.2014.166.issue-3, PMID: 24673627

[15] Mitelman F Mertens F. Mitelman database of chromosome aberrations and gene fusions in cancer. (2025). J. B.

[16] Guan W Zhou L Li Y Yang E Liu Y Lv N. Profiling of somatic mutations and fusion genes in acute myeloid leukemia patients with FLT3-ITD or FLT3-TKD mutation at diagnosis reveals distinct evolutionary patterns. Exp Hematol Oncol. (2021) 10:27. doi: 10.1186/s40164-021-00207-4, PMID: 33836835 PMC8033687

[17] Dong S Chen J. SUMOylation of sPRDM16 promotes the progression of acute myeloid leukemia. BMC Cancer. (2015) 15:893. doi: 10.1186/s12885-015-1844-2, PMID: 26559765 PMC4641379

